# Society Meetings

**Published:** 1902-03

**Authors:** 


### SOCIETY MEETINGS.

The Buffalo Academy of Medicine held meetings during the months of January and February, 1902, as follows:

*Section on Obstetrics.*—Tuesday evening, January 28. Program: In pelvic suppuration, when shall we operate by the abdomen, and when by the vagina? William R. Pryor, Prof. of Gynecology, New York Polyclinic.

*Section on Surgery.*—Tuesday evening, February 4. Program: The significance of the use of Credé's silver preparations in surgery, Roswell Park. The blood and its relation to surgical diagnoses, Albert E. Woehnert.

*Section on Medicine.*—Tuesday evening, February 11. Program: Pneumococci and cardiac toxemia, Henry L. Elsner, Syracuse; Baby farming in Buffalo, Julius Pohlman.

*Ophthalmology, Otology, Laryngology and Rhinology.*—Monday evening, February 17. Program: The treatment of empyema of the sphenoid sinus, F. W. Hinkel.

*Section on Pathology.*—Tuesday evening, February 18. Program: Clinical and pathological cases in dermatology (stereopticon) John Addison Fordyce, Professor of dermatology and syphilography, University and Bellevue Medical College, New York City.

*Section on Obstetrics.*—Tuesday evening, February 25. Program: Interesting experiences in abdominal surgery, Carlton C. Frederick. Retroversions in girls and unmarried women, Herman E. Hayd.

---

The Harvard Medical Society, of New York City, at its annual meeting, held at the Metropolitan Club on Saturday evening, January 24, 1902, elected the following-named officers for the current year: President, William B. Coley; vice-president, Myron P. Denton; secretary, J. Hilton Waterman; treasurer, Joseph A. Kenefick.

---

The Physicians' League held its fourth meeting for the season 1901-1902 at the Buffalo State Hospital, January 27, 1902, the guest of Dr. Helene Kuhlman. Dr. Willis G. Gregory was the essayist of the evening and, by invitation, read an interesting paper on The pharmacopœia.

